# Supplementary material for: Crystal Structure of the Cyclostreptin-Tubulin Adduct: Implications for Tubulin Activation by Taxane-Site Ligands
Source: Int J Mol Sci. 2019 Mar 20;20(6):1392. doi: 10.3390/ijms20061392 (PMC6471726; doi:10.3390/ijms20061392)
Supplement: Supplementary file 1 [file ijms-20-01392-s001.pdf]

Crystal structure of the cyclostreptin-tubulin adduct: Implications for tubulin activation by taxane-site ligands.

Francisco de Asís Balaguer[1]; Tobias Mühlethaler [2]; Juan Estevez-Gallego[1]; Enrique Calvo[3]; Juan Francisco Giménez-Abián[1]; April L. Risinger[4]; Erik J. Sorensen[5]; Cristopher D. Vanderwal[6]; Karl-Heinz Altmann[7]; Susan L. Mooberry[4]; Michel O. Steinmetz[2,8]; María Ángela Oliva[1]; Andrea E. Prota*[2]; J. Fernando Díaz*[1].

[1]Structural and Chemical Biology Department. Centro de Investigaciones Biológicas, CSIC, Ramiro de Maeztu 9, 28040 Madrid, Spain.

[2]Laboratory of Biomolecular Research, Division of Biology and Chemistry, Paul Scherrer Institut, 5232 Villigen PSI, Switzerland.

[3]Unidad de Proteómica. Centro Nacional de Investigaciones Cardiovasculares, CNIC. Madrid, Spain.

[4]Department of Pharmacology, The University of Texas Health Science Center at San Antonio, San Antonio, Texas, 78229-3900, USA.

[5]Department of Chemistry, Princeton University, Princeton, New Jersey 08544, United States

[6]Department of Chemistry, 1102 Natural Sciences II, University of California, Irvine, California 92697-2025, USA

[7]ETH Zürich, Department of Chemistry and Applied Biosciences, Institute of Pharmaceutical Sciences, Zürich, Switzerland.

[8]University of Basel, Biozentrum, 4056 Basel, Switzerland.

*Correspondence may be addressed to JFDP and AEP: fer@cib.csic.es; andrea.prota@psi.ch

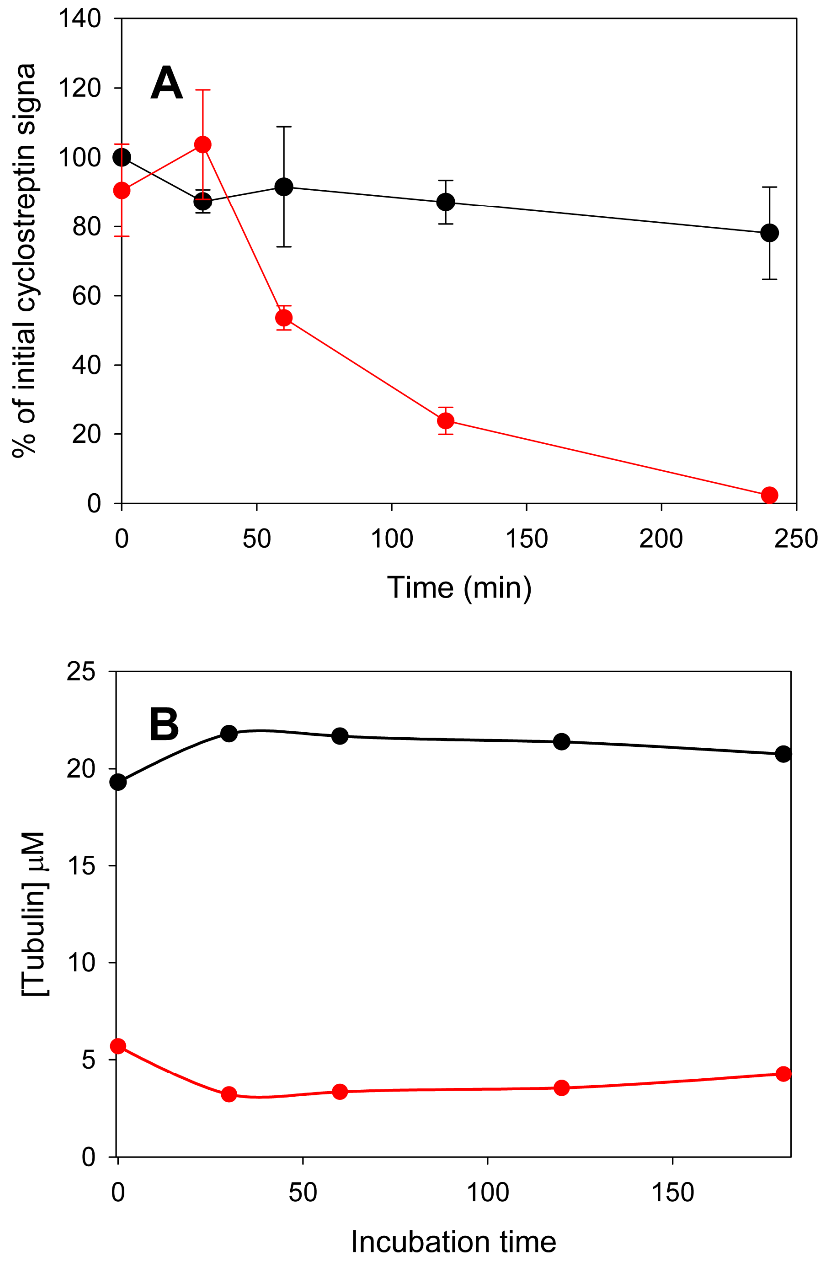

Figure S1. Cyclostreptin binding and stability of tubulin-cyclostreptin adduct : A). Time course of reaction of 25μM cyclostreptin with dimeric tubulin followed by HPLC-MS. Black circles and lines: Unreacted cyclostreptin in the absence of tubulin. Red circles and lines: Unreacted cyclostreptin in the presence of 20 μM tubulin. B) Quantification of the assembly of the tubulin-cyclostreptin complex incubated at different times. Black circles and lines pelleted tubulin (microtubules), red circles and lines supernatant tubulin (not assembled dimers).

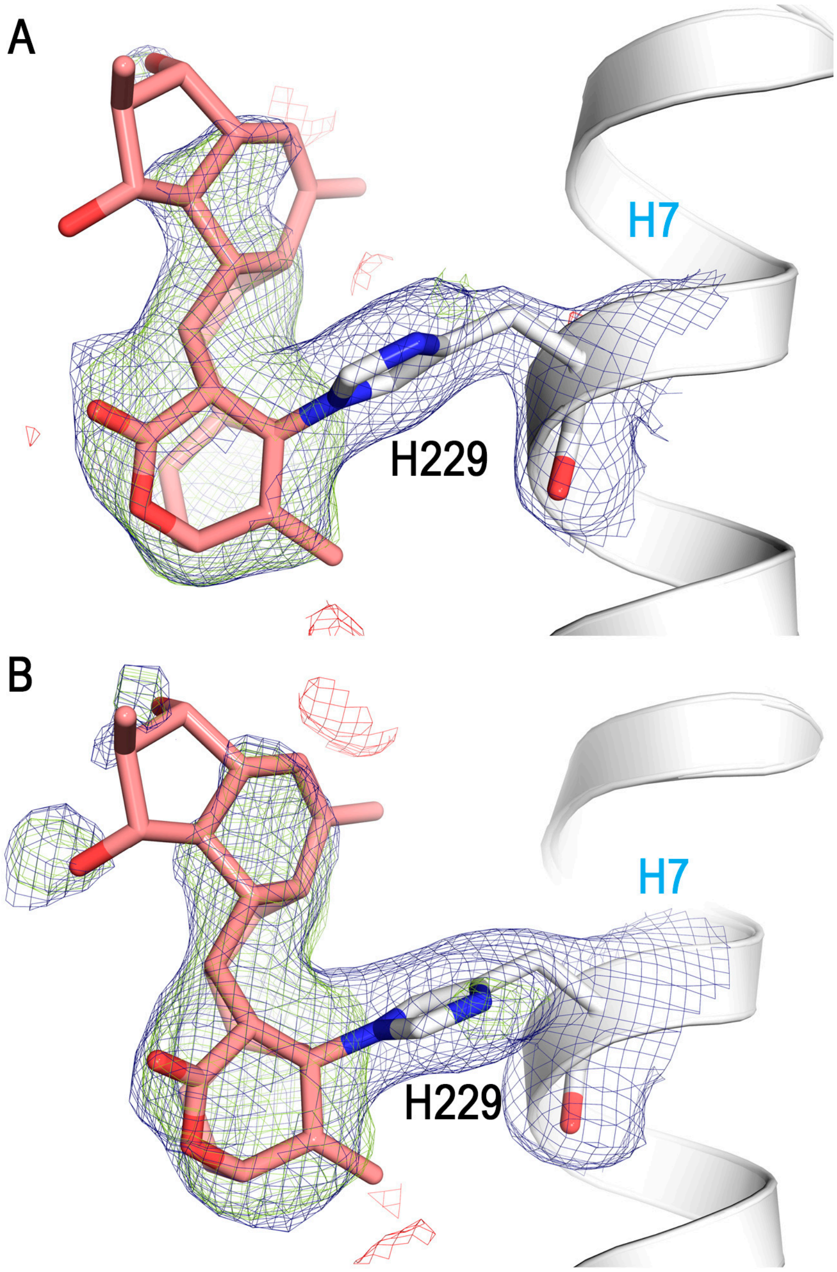

Figure S2. Electron-density maps of both the cyclostreptin molecules bound to tubulin in chain B (panel A) and chain D (panel B) of the T_2R-TTL complex. The SigmaA-weighted 2mFo-DFc (dark blue mesh contoured at +0.7σ) and mFo-DFc (light green and red mesh contoured at +/- 2.5σ, respectively) simulated annealing omit maps were calculated by excluding the atoms of the cyclostreptin molecules. Both the covalently bound cyclostreptin molecules (salmon) and His229 residues are depicted in stick representation.

Table S1. Data collection and refinement statistics for the T_2R-TTL-cyclostreptin complex

	T2R-TTL-cyclostreptin
**Data collection**	
Space group	$P2_12_12_1$
Cell dimensions	
a, *b*, *c* (Å)	104.6, 158.4, 179.95
Resolution (Å)	49.7 – 1.9 (1.95-1.90)
R_{merge} (%)	10.2 (448.5)
R_{meas} (%)	10.4 (457.2)
R_{pim} (%)	2.5 (84.3)
$I / \sigma I$	22.7 (0.9)
CChalf	100 (32.2)
Completeness (%)	100 (100)
Redundancy	26.8 (27.0)
**Refinement**	
Resolution (Å)	49.7 – 1.9
No. unique reflections	234314
R_{work} / R_{free}	18.7 / 21.3
No. atoms	
Protein	17404
Ligand	58
Water	681
Average *B*-factors (Å^2)	
Protein	62.3
Ligand (chain B / D)	85.1 / 91.5
Water	55.7
Wilson *B*-factor	42.4
R.m.s. deviations	
Bond lengths (Å)	0.004
Bond angles (°)	0.652
Ramachandran statistics[c]	
Favored regions (%)	98.0
Allowed regions (%)	2.0
Outliers (%)	0

*Values in parentheses are for highest-resolution shell.
